# Sex Differences in the Default Mode Network with Regard to Autism Spectrum Traits: A Resting State fMRI Study

**DOI:** 10.1371/journal.pone.0143126

**Published:** 2015-11-24

**Authors:** Minyoung Jung, Maria Mody, Daisuke N. Saito, Akemi Tomoda, Hidehiko Okazawa, Yuji Wada, Hirotaka Kosaka

**Affiliations:** 1 Research Center for Child Mental Development, University of Fukui, Eiheiji, Fukui, Japan; 2 Athinoula A. Martinos Center for Biomedical Imaging, Department of Radiology, Massachusetts General Hospital, Charlestown, MA, United States of America; 3 Developmental Emotional Intelligence, Division of Developmental Higher Brain Functions, Department of Child Development United Graduate School of Child Development, Osaka University, Kanazawa University, Hamamatsu University School of Medicine, Chiba University and University of Fukui, Eiheiji, Fukui, Japan; 4 Biomedical Imaging Research Center, University of Fukui, Eiheiji, Fukui, Japan; 5 Department of Neuropsychiatry, Faculty of Medical Sciences, University of Fukui, Eiheiji, Fukui, Japan; University Of Cambridge, UNITED KINGDOM

## Abstract

Autism spectrum traits exist on a continuum and are more common in males than in females, but the basis for this sex difference is unclear. To this end, the present study draws on the extreme male brain theory, investigating the relationship between sex difference and the default mode network (DMN), both known to be associated with autism spectrum traits. Resting-state functional magnetic resonance imaging (MRI) was carried out in 42 females (mean age ± standard deviation, 22.4 ± 4.2 years) and 43 males (mean age ± standard deviation, 23.8 ± 3.9 years) with typical development. Using a combination of different analyses (viz., independent component analysis (ICA), fractional amplitude of low-frequency fluctuation (fALFF), regional homogeneity (ReHo), and seed-based analyses), we examined sex differences in the DMN and the relationship to autism spectrum traits as measured by autism-spectrum quotient (AQ) scores. We found significant differences between female and male subjects in DMN brain regions, with seed-based analysis revealing a significant negative correlation between default-mode resting state functional connectivity of the anterior medial prefrontal cortex seed (aMPFC) and AQ scores in males. However, there were no relationships between DMN sex differences and autism spectrum traits in females. Our findings may provide important insight into the skewed balance of functional connectivity in males compared to females that could serve as a potential biomarker of the degree of autism spectrum traits in line with the extreme male brain theory.

## Introduction

Autism spectrum disorder (ASD) is a neurodevelopmental disorder characterized by qualitative deficits in social communication and social interaction and unusually restricted, repetitive behaviors and interests [1,2]. These characteristics, so-called “autism spectrum traits,” exist on a continuum that extends between individuals with ASD and individuals with typical development (TD) [3–5]. Moreover, ASD is more common in males than in females [6–8], but the reason for this sex difference is unclear. One possible explanation put forth by the extreme male brain theory posits that autism spectrum traits are associated with the extreme characteristics of the male brain, specifically “less empathizing” and “more systemizing” characteristics compared to females [9]. Investigations into the extreme male brain theory have provided evidence regarding the autism spectrum traits in individuals regardless of ASD diagnosis. For example, researchers have found that males in the general population show higher scores for autism spectrum traits (measured using the autism-spectrum quotient[AQ]) than females and usually have the highest individual ASD scores of all participants [10]. Moreover, males score lower than females on measures relevant to autism spectrum traits, such as the empathy quotient (EQ), reading the mind in the eyes test, and social sensitivity [11,12]. Taken together, these findings indicate that it is important to evaluate sex differences in the general population for the quantitative support of daily difficulties associated with autism spectrum traits in each individual, regardless of ASD diagnosis.

Neuroimaging evidence supporting the extreme male brain theory has shown that the male brain reveals a skewed balance between local and inter-hemispheric connectivity compared to the female brain [13,14]. The level of brain activation also varies between females and males as a function of autism spectrum traits, evident on tasks of social cognition such as social reward, biological motion, self-cognition, and facial expressions, in brain regions including medial prefrontal cortex (MPFC), and posterior cingulate cortex (PCC) [15–17]. Interestingly, MPFC and PCC overlap with the default mode network (DMN) involved in social cognition including mentalizing and empathizing, which are classically related to autism spectrum traits, based on resting-state functional magnetic resonance imaging (rs-fMRI) [18,19]. Rs-fMRI has emerged as a useful tool for investigating intrinsic fluctuations in blood oxygen level-dependent (BOLD) signals that may provide a window into more fundamental neuronal signatures in the DMN [20,21]. A powerful advantage of rs-fMRI is that it does not involve doing a task and hence, is free of activation effects unrelated to the task especially in clinical populations that have behavioral and compliance issues (e.g. attention, gaze) [22–24]. Moreover, rs-fMRI studies in males with and without ASD have revealed that autism spectrum traits are associated with hypoconnectivity in DMN brain regions including MPFC and PCC [25,26]. Thus, investigating the relationship between sex differences and DMN in relation to autism spectrum traits in neurotypical controls may help illuminate the brain basis of these traits.

While rs-fMRI studies in individuals with and without ASD have suggested that the DMN is associated with autism spectrum traits, the relationship to sex differences has not been clearly established. Only a few studies have reported significant differences between females and males in the DMN, mostly using independent component analysis (ICA) [27,28]. Although the ICA approach provides information about functional connectivity of a resting state network [22,29–31], it does not provide information about local connectivity and the synchronicity of spontaneous activity among brain regions in DMN [32–36]. Thus, more work is needed on how these measures may jointly advance our understanding about functional connectivity.

Fractional amplitude of low-frequency fluctuation (fALFF) analysis and regional homogeneity (ReHo) analysis are relatively new approaches, which provide complementary information about spontaneous brain activity and local connectivity within the resting-state DMN[33,35,37,38]. fALFF can weaken biases of physiological noise and thus provide information about the power of spontaneous low frequency fluctuations relative to all frequencies within DMN during resting state [33,39]. ReHo provides information of the local synchronization of the BOLD signal based on Kendall's correlation coefficient (KCC) between time series of a given voxel and its neighbors [35,38,40]. Thus, resting-state data lends itself to analysis in a variety of ways to effectively measure more fundamental neuronal signatures of the DMN. To the best of our knowledge, no studies have evaluated sex differences of the DMN in young adult subjects in the context of autism spectrum traits, which are thought to be associated with localized brain regions and resting-state functional connectivity between them.

In the present study, we use rs-fMRI to examine sex differences in the DMN with regard to autism spectrum traits in typically developing young adults (TD), females and males, matched on age. We draw on several analytical methods to provide complementary information, including ICA to determine the brain regions affected by sex differences in the DMN, fALFF analysis to determine the spontaneous signal, ReHo analysis to reveal locally synchronized spontaneous brain activity, and seed-based analysis to identify implicated brain regions and connections in the DMN. Based on the extreme male brain theory, we predicted that resting state connectivity in key DMN brain regions (e.g., MPFC and PCC) would be negatively correlated with AQ scores in the male group but not in the female group.

## Materials and Method

### Participants

A total of 87 right-handed, healthy young adult participants (42 females, 45 males) were recruited for the current study by the Department of Neuropsychiatry of the University of Fukui Hospital, Japan. The two groups, males and females, were matched on age with no significant difference between them (*p* = 0.12). To quantify participants’ autistic traits, we used the AQ scores, a validated measure of autism spectrum traits found within both the typical population and individuals diagnosed with ASD, the 50 items were divided into five theoretically derived subscales of 10 items each: Social skill; Communication; Imagination; Attention to detail; and Attention switching [10]. There was a significant difference between the male and female groups for imagination scores (*p* < 0.01) but not for other subscale scores and total AQ scores. Participants were excluded if they had high total AQ scores (over 33) [10] or a history of major medical or neurological illness (“e.g., epilepsy or significant head trauma) or a lifetime history of alcohol or drug dependence using the Structured Clinical Interview for the Diagnostic and Statistical Manual of Mental Disorders Axis I (SCID I) and Axis II disorders (SCID II). The protocol used for this study was approved by the ethics committee of the University of Fukui. After a complete explanation of the study, all participants provided written, informed consent.

#### Image acquisition

Functional images were acquired with a T2*-weighted gradient-echo echo-planar imaging (EPI) sequence with a 3-T imager (Discovery MR 750; General Electric Medical Systems, Milwaukee, WI, USA) and a 32-channnel head coil. A total of 201 volumes were acquired for a total imaging time of 7 min 42 s. Each volume consisted of 40 slices, with a thickness of 3.5 mm and a 0.5-mm gap to cover the entire brain. The time interval between each successive acquisition of the same slice (repetition time, TR) was 2300 ms, with an echo time (TE) of 30 ms and a flip angle (FA) of 81°. The field of view (FOV) was 192 × 192 mm, and the matrix size was 64 × 64, yielding volume dimensions of 3 × 3 mm. The participants were instructed to stay awake but close their eyes and think of nothing in particular. Participant movement was further minimized by the placement of memory-foam pillows around their head.

### fMRI data preprocessing

fMRI data were analyzed using SPM8 (http://www.fil.ion.ucl.ac.uk/spm/), a data processing assistant, and resting-state fMRI software (DPARSF) [41] with the following steps. First, the initial 10 volumes were discarded, and slice-timing correction was performed, followed by spatial realignment of 191 volumes to the mean volume. The signal from each slice was realigned temporally to that obtained from the middle slice using sinc interpolation. The re-sliced volumes were normalized to the Montreal Neurological Institute (MNI) space with a voxel size of 2 × 2 × 2 mm using the EPI template provided by SPM8. The normalized images were spatially smoothed with a 6-mm Gaussian kernel (except for ReHo; Smoothing process was performed after ReHo analysis) [35,42]. Next, the linear trend in the time series was removed, and temporal bandpass filtering (0.01–0.08 Hz, except for fALFF) was performed to reduce the effects of low-frequency drift and high-frequency noise [43]. The non-neural noise in the time series was controlled, and several sources of spurious variance (e.g., the Friston 24-parameter model, white matter signals, and cerebrospinal fluid signals) were removed from the data through linear regression [44,45].

To control for motion confounds in our data, we investigated the effects of head motion by computing the mean frame-to-frame root mean squared (RMS) motion, and frame-wise displacement (FD) obtained during the realignment process. Two participants (2 males) had to be removed from the analysis due to exclusion criteria of excessive head motion (over 2.5 mm, 2.5 degree, and 0.15 mean FD) in the scanning. As a result, data from 85 right-handed, healthy participants (42 females, 43 males) were included in the analyses (Table 1). There were no significant group differences in mean frame-to-frame RMS motion (*p* = 0.77) [46] or frame-wise displacement (*p* = 0.46) [47].

**Table 1:** Demographic data of the participants.

Measure	Female	Male	*p* value
	(n = 42)	(n = 43)	
Age (SD)	22.4 (4.2)	23.8 (3.9)	0.12
(range)	(18–39)	(19–35)	
Handedness: Right / Left^a^	42/0	43/0	-
RMS Mean Displacement (mm)	0.03	0.03	0.77
SD	0.01	0.01	
Mean FD (mm)	0.13	0.13	0.46
SD	0.05	0.04	
Autism spectrum traits			
Total AQ (SD)	15.0 (6.5)	17.5 (5.8)	0.07
Social interaction (SD)	11.3 (6.1)	13.7 (5.2)	0.06
Social skills (SD)	2.5 (2.3)	3.3 (1.9)	0.11
Communication (SD)	2.7 (2.3)	2.5 (1.9)	0.66
Attention switching (SD)	4.1 (2.1)	4.4 (1.8)	0.59
Imagination (SD)	1.9 (1.1)	3.5 (1.6)	< 0.001*
Attention to detail (SD)	3.6 (2.3)	3.8 (1.9)	0.84

AQ, autism spectrum quotient; FD, frame-wise displacement; RMS, root mean square;

^a^ Assessed by the Edinburgh handedness inventory.

All participants were right-handed.

*p < 0.01, with independent-samples t-test.

### Data Analysis

#### ICA

Group spatial ICA was performed using the Group ICA/IVA of fMRI Toolbox (GIFT, http://icatb.sourceforge.met/, version 2.0 a) based on the FAST ICA algorithm [48]. To estimate spatial maps and time courses in the DMN accurately, the analysis involved several steps using group ICA [49]: (1) a mean of 30 components was analyzed for each subject; (2) data reduction used principal components analysis followed by independent component estimation that produced spatial maps and time courses with FAST ICA [50]; (3) the group ICA was calculated by back reconstruction including dual regression for each participants; (4) the offset was computed using the mean component maps, which were subtracted from the subject component maps; and (5) the mean ICA component were standardized into Z-scores across participants; (6) we performed group ICA in females and males separately to confirm that the same components were present in each group. The spatial template matching procedure was carried out using the DMN template provided in the GIFT toolbox. Two components were selected based on ranked highest correlation values (component A: 0.52, component B: 0.44) and matched brain regions associated with the DMN. These two components were identifies as the anterior and posterior DMN in a previous study [51]. For group comparisons, we first extracted the Z map for the total group (combining female and male) using a one-sample t-test (thresholded at *p* = 0.05 corrected for family-wise error) from two components and then examined differences in DMNs using a two-sample t-test (female versus male), thresholded at *p* < 0.001 uncorrected for height and a cluster *p* < 0.05 uncorrected for extend. Results of two-sample t-tests were masked by the results of one-sample t-tests (thresholded at *p* < 0.001 uncorrected for height and a cluster *p* < 0.05 uncorrected for extend) [52].

#### Regional measures

In fALFF nalaysis, each time series at each voxel as the fraction was calculated between the sum of amplitudes of the band ranging from 0.01 to 0.08 Hz, and subject-level voxel-wise fALFF maps were standardized into subject-level Z-score maps using the DPARSF toolbox. For group comparisons, each image pertaining to z values reflecting fALFF was entered into two-sample t-tests, set thresholded at *p* < 0.001 uncorrected for height and a cluster *p* < 0.05 uncorrected for extend. To focus the DMN, we used masks generated from results of the one-sample t-tests (thresholded at *p* < 0.001 uncorrected for height and a cluster *p* < 0.05 uncorrected for extend) [53].

ReHo analysis was also performed using the DPARSF toolbox. Individual ReHo Z-score maps were created by subtracting the mean value for the entire brain from each voxel and dividing by the corresponding standard deviation. ReHo is computed as the Kendall's correlation coefficient (KCC) between the time course of that voxel and its 26 neighboring voxels [35]. The mean frame-to-frame RMS motion and mean FD were included as covariates in the model due to the potential for spurious signal changes. Edge voxels were excluded from analyses [35]. To focus the DMN, we used masks based on the results of the one-sample t-tests (thresholded at *p* < 0.001 uncorrected for height and a cluster *p* < 0.05 uncorrected for extend) [54].

#### Seed-based rs-FC analysis

To allow comparison of our findings with those from ASD-related DMN studies, we conducted seed-based rs-FC analysis focusing on regions in which young adults with and without ASD show significant differences in the DMN [25,55–57]. The seed regions including the aMPFC (MNI coordinates x = -6, y = 52, z = -2) and PCC (MNI coordinates x = -8, y = -56, z = 26) were selected from DMN meta-analysis in accordance with previous studies [19]. The mean time course of all voxels in each seed measuring 8 mm in radius was used to calculate voxel-wise linear correlations (Pearson’s correlations) for the whole brain, and individuals’ r values were then normalized to z values using Fisher’s z transformation. For group comparisons, we first extracted the z map for the total group (both sexes) in a one-sample t-test (thresholded at *p* = 0.05 corrected for family-wise error) and then examined differences in the DMN in a two-sample t-test (female versus male), set thresholded at *p* < 0.001 uncorrected for height and a cluster *p* < 0.05 uncorrected for extend [25]. The mean frame-to-frame RMS motion and frame-wise displacement were included as covariates in the model due to the potential for spurious signal changes [58].

#### Correlation with autism spectrum traits

We ran correlation analyses to investigate the relationship between group differences obtained in the ICA, fALFF, ReHo, and seed-based analysis and autism spectrum traits separately for each group, removing the effects of age using partial correlations. Measures of autism spectrum traits were calculated using AQ scores, which included total AQ scores and the social interaction score [59].

## Result

### Demographic and clinical characteristics

Demographic and clinical characteristics of the study participants are presented in Table 1. In an earlier study of total AQ scores and imagination scores in a Japanese sample, the authors found a sex difference with males scoring higher than females (*p* < 0.10) [60]. Our finding regarding the total AQ score in males vs. females (*p* = 0.07) was at the same statistical level. In a separate study, a sample from the UK also showed typical males to score higher in AQ including imagination scores than typical females, providing further support for this psychometric profiles of AQ in a large-scale sample [10].

### ICA

**Fig 1:**
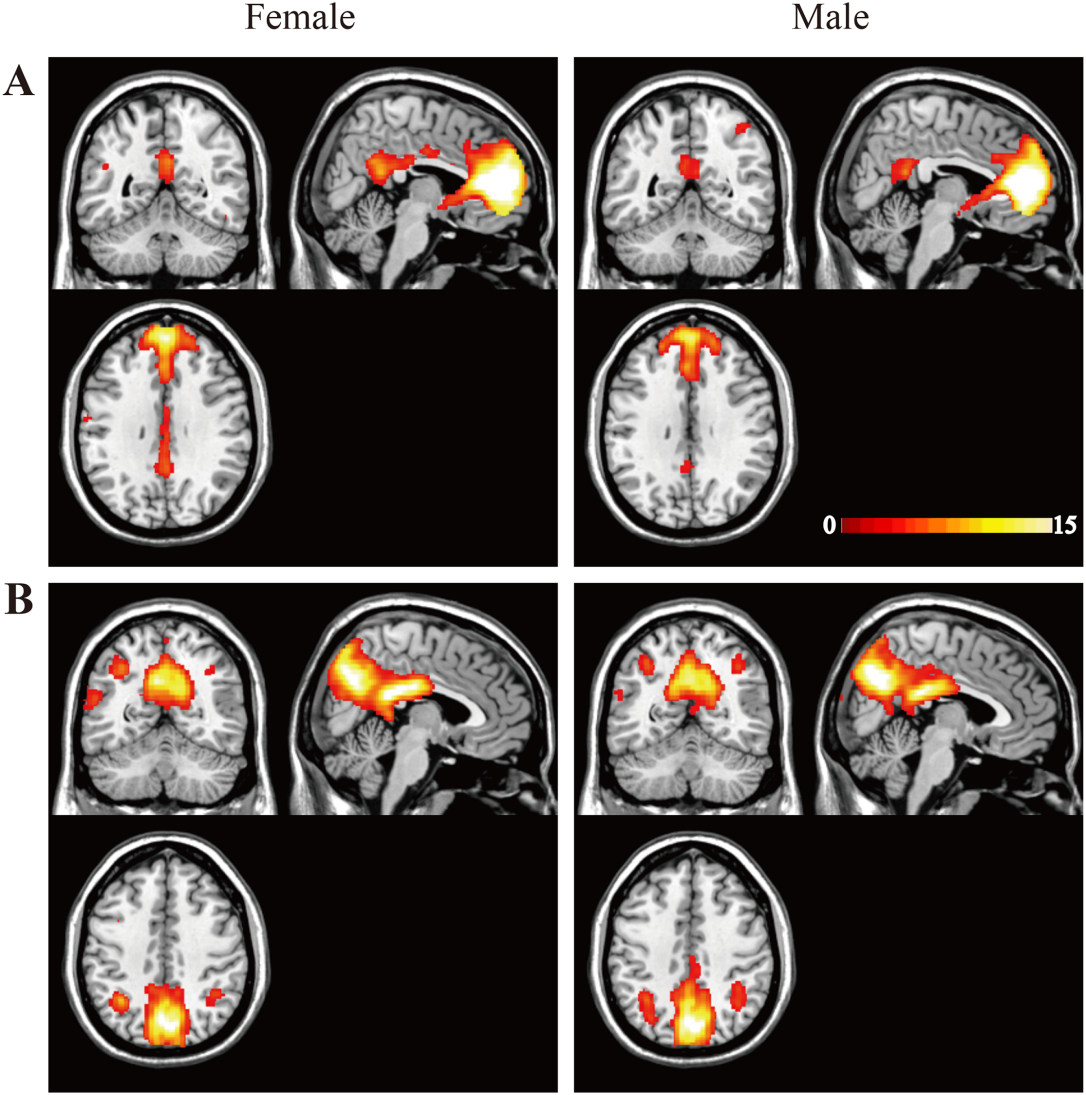
Independent component analysis (ICA) results. The average components for females and males are shown in the left and right columns, respectively. (A) Independent components of the anterior default mode network (DMN). (B) Independent components of posterior DMN. The maps depict the statistical threshold for both results set at *p* = 0.05 with whole-brain FWE correction. The coordinates for the panels above are (A) (4, -52, 32) and (B) (4, -58, 42). Color bars denote the t-statistic range.

We found two components corresponding to the DMN in each group, including the anterior and posterior DMN (Fig 1). Within the anterior DMN (Fig 1A), both females and males showed significant functional activation of the MPFC (MNI coordinates x = 4, y = 48, z = 6; *p* = 0.05 with FWE correction) and PCC (MNI coordinates x = 4, y = -48, z = 20; *p* = 0.05 with FWE correction), with no significant differences in brain regions between the two groups. Within the posterior DMN (Fig 1B), both females and males showed significant functional activation within the PCC (MNI coordinates x = 4, y = -36, z = 24; *p* = 0.05 with FWE correction) and MPFC (MNI coordinates x = 6, y = 36, z = 16; *p* = 0.05 with FWE correction), and there were no significantly different brain regions between the two groups.

### Regional measures

**Fig 2:**
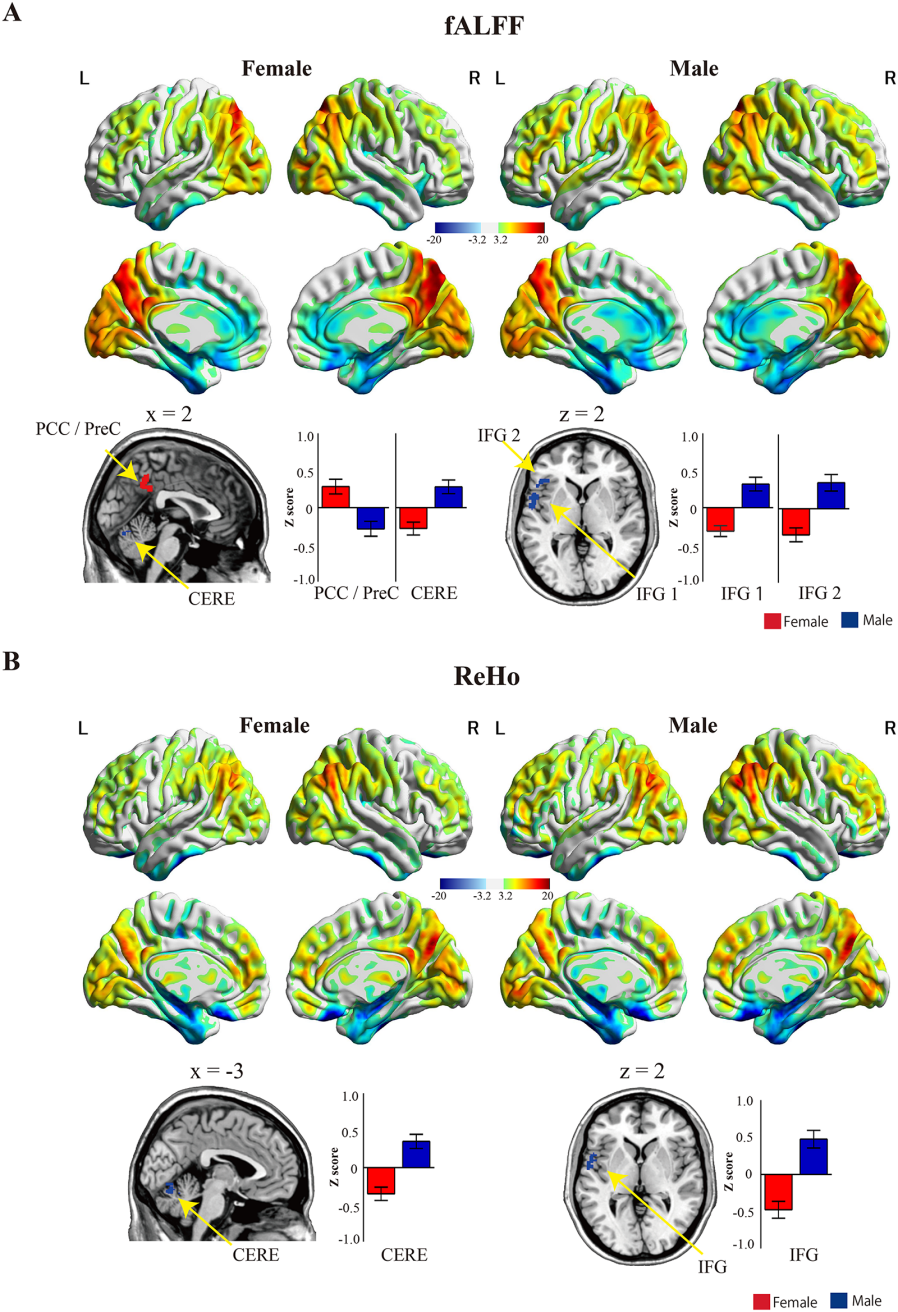
Regional measure results. (A) fALFF Z maps for females and males are shown on the left and right, respectively. Females showed significantly stronger fALFF results in the PCC/PreC compared with males, but males fALFF results were stronger in the CERE and IFG. The maps depict the statistical threshold at *p* < 0.001 uncorrected for height and a cluster at *p* < 0.05 uncorrected for extend. Color bars denote the t-statistic range. CERE, cerebellum; IFG, inferior frontal gyrus; L, left; PCC, posterior cingulate cortex; PreC, precuneus; R, right. (B) ReHo fALFF Z maps for females and males are shown on the left and right, respectively. Males showed significantly stronger ReHo results in the CERE and IFG compared with females. The maps depict the statistical threshold at *p* < 0.001 uncorrected for height and a cluster at *p* < 0.05 uncorrected for extend. The bar graph indicates the mean and standard error of Z scores in each group. The red and blue bars indicate females and males, respectively. Colored bars denote the t-statistic range. CERE, cerebellum; fALFF, fractional amplitude of low-frequency fluctuation; IFG, inferior frontal gyrus; L, left; PCC, posterior cingulate cortex; R, right; ReHo, regional homogeneity.

The fALFF and ReHo analysis revealed different results for the two groups in anterior and posterior DMN. In the two-sample t-test, females showed stronger fALFF in the PCC/precuneus (PreC) compared with males. Males, however, showed stronger fALFF in the cerebellum and inferior frontal gyrus (IFG) compared to females, but only in anterior DMN (Table 2 and Fig 2A). The ReHo analysis yielded no sex differences except in the IFG and cerebellum in anterior DMN where males showed more synchronized activity than females. (Table 2 and Fig 2B).

**Table 2:** Regional measures of intrinsic functional architecture in the DMN in male and female groups.

Region	Side	MNI coordinates			Z-score	Cluster size
		x	y	z		*kE* (voxels)
**Anterior DMN mask**						
**fALFF**						
**Female > Male**						
Posterior cingulate cortex / Precuneus	L	2	-52	38	3.65	49
**Male > Female**						
Inferior frontal gyrus	L	-48	24	2	4.70	42
Inferior frontal gyrus	L	-56	-2	2	4.22	107
Cerebellum	L	-2	-68	-14	3.94	36
**ReHo**						
**Female > Male**						
None						
**Male > Female**						
Inferior frontal gyrus	L	-56	-2	2	4.98	66
Cerebelum	L	-4	-70	-12	4.70	120
**Posterior DMN mask**						
**fALFF**						
**Female > Male**						
Posterior cingulate cortex / Precuneus	L	2	-52	38	3.65	149
**Male > Female**						
None						
**ReHo**						
**Female > Male**						
None						
**Male > Female**						
None						

The statistical threshold for contrasts was *p* <0.001 uncorrected for height and cluster *p* <0.05 uncorrected for extend.DMN, default mode network; fALFF, fractional amplitude of low-frequency fluctuations; MNI, Montreal Neurological Institute; ReHo, regional homogeneity.

### Seed-based functional connectivity

**Fig 3:**
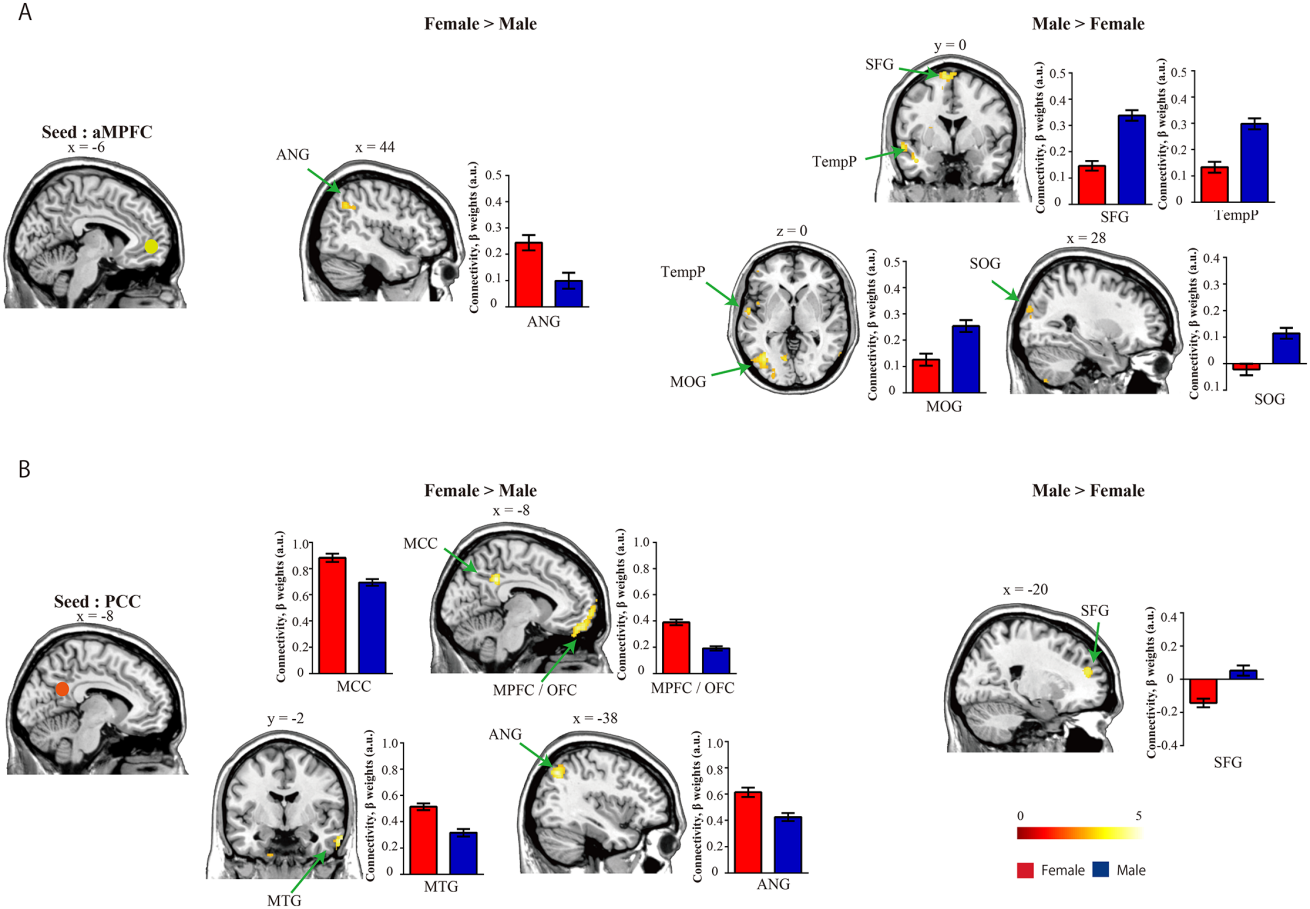
Seed-based analysis functional connectivity results. (A) Comparison of aMPFC seed ROI functional connectivity between groups. Females showed stronger connectivity to the ANG, but males showed stronger connectivity to the SFG, TempP, MOG, and SOG. (B) Comparison of PCC seed ROI functional connectivity. Females showed stronger connectivity to the MOG, MPFC/OFC, MTG, and ANG, but males exhibited stronger connectivity in the SFG. The maps depict the statistical threshold at *p* < 0.001 uncorrected for height and a cluster at *p* < 0.05 uncorrected for extend. The bar graph indicates the mean and standard error of beta weights in each group. The red and blue bars indicate females and males, respectively. Color bars denote the t-statistic range. aMPFC, anterior prefrontal cortex; ANG, angular gyrus; MOG, middle occipital gyrus; MPFC, medial prefrontal cortex; MTG, middle temporal gyrus; OFC, orbital frontal cortex. PCC, posterior cingulate cortex; SFG, superior frontal gyrus; TempP, temporal pole.

Females showed stronger connections between the aMPFC seed and angular gyrus (ANG) compared with males (Table 3 and Fig 3A). In contrast, males showed stronger connections from the aMPFC seed to the superior frontal gyrus (SFG), temporal pole (TempP), middle occipital gyrus (MOG), and superior occipital gyrus (SOG) (Table 3 and Fig 3A). In the PCC, female showed stronger connections compared to the males from the PCC seed to middle temporal gyrus (MTG), MPFC/orbital frontal cortex (OFC), middle cingulate cortex (MCC), and ANG (Table 3 and Fig 3B). In contrast, males showed higher connectivity than females from the PCC seed to SFG.

**Table 3:** Significant differences in functional connectivity between groups from the seed-based analysis.

Region	Side	MNI coordinates			Z-score	Cluster size
		x	y	z		*kE* (voxels)
**seed: aMPFC**						
**Female > Male**						
Angular gyrus	R	44	-56	38	3.59	99
**Male > Female**						
Superior frontal gyrus	L	-4	8	70	4.84	1641
Temporal pole	L	-48	8	-14	4.77	610
Middle occipital gyrus	L	-38	-70	0	4.23	1180
Superior occipital gyrus	L	24	-90	28	3.83	235
**seed: PCC**						
**Female > Male**						
Middle temporal gyrus	R	60	-2	-32	4.72	152
Medial prefrontal cortex / Orbital frontal cortex	L	-4	56	-20	4.70	951
Middle cingulate cortex	L	-8	-44	36	4.50	219
Angular gyrus	L	-38	-66	44	4.49	161
**Male > Female**						
Superior frontal gyrus	L	20	48	22	4.16	231

The statistical threshold for contrasts was *p* <0.001 uncorrected for height and cluster *p* <0.05 corrected for extend. aMPFC, anterior medial frontal cortex; PCC, posterior cingulate cortex; MNI, Montreal Neurological Institute.

### Correlation with autism spectrum traits

**Fig 4:**
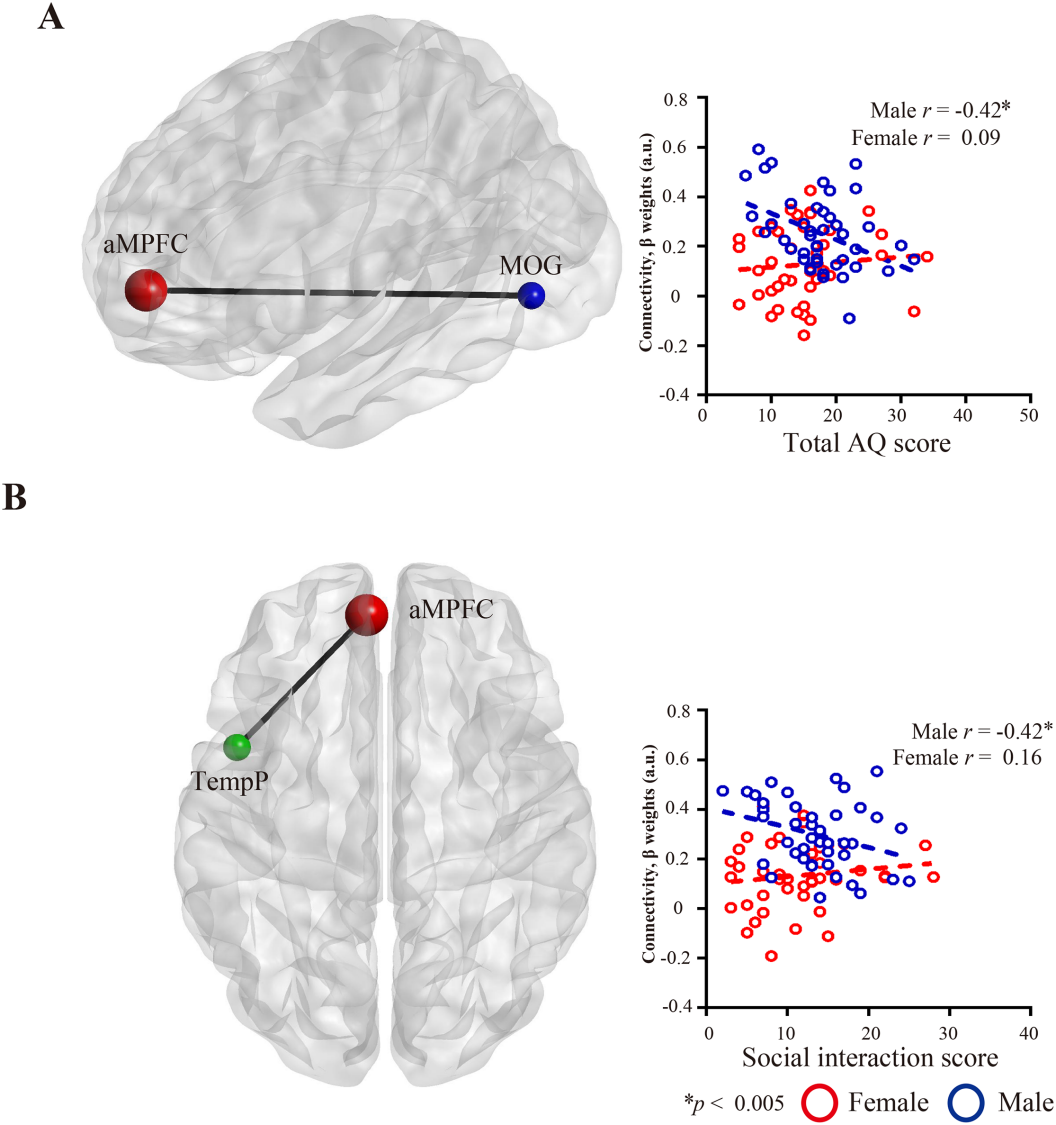
Correlations between autism spectrum traits and functional connectivity. (A) Functional connectivity between the aMPFC and MOG were negatively correlated with total AQ score in males not in females. (B) Functional connectivity between the aMPFC and TempP were negatively correlated with functional connectivity strength and social interaction scores in males not in females. The red and blue circles represent females and males, respectively. aMPFC, anterior prefrontal cortex; MOG, middle occipital gyrus; rs-FC, resting state functional connectivity; TempP, temporal pole.

In the female group, there were no significant correlations between group differences in the DMN and total AQ scores and social interaction scores. In contrast, the male group showed that AQ total scores were significantly negatively correlated with functional connectivity between the aMPFC and MOG (Fig 4A), which showed stronger connections in seed-based analysis in the males compared to females. The male group also showed significant negative correlations between AQ social interaction scores and rs-FC between aMPFC and TemP (Fig 4B), where once again males showed stronger connections in seed-based analysis compared to females. Group differences in the fALFF, and ReHo, however, were not correlated with autism spectrum traits in either group.

## Discussion

In the present study, we utilized rs-fMRI and AQ scores to investigate sex differences in DMN with regard to autism spectrum traits in the general population. Compared to the female group, the male group showed stronger seed-based resting state connectivity in keys areas of the DMN including the aMPFC and PCC. Furthermore, the male group showed negative correlation between functional connectivity of the aMPFC seed (with MOG and TemP), and autism spectrum traits. These results suggest that connectivity differences in the DMN might affect males more often than females with regard to autism spectrum traits.

### Gender-related differences within the DMN

In the present study, we found that female group showed higher fALFF in the PCC compared to the male group. The PCC along with the MPFC on the midline of brain comprise a core region within the DMN [18,19]. Neuroimaging evidence has shown that females show heightened activation in the PCC during the working memory tasks entailing cognitive control of negative emotions [61]. As such, the higher fALFF in PCC in the female than male group in the present study may reflect a gender-specific pattern of the DMN.

In contrast to females, males showed increased fALFF and ReHo in the IFG and cerebellum. In other studies, males have shown stronger activation in the IFG and cerebellum on a self-observation of social stress task [16] and in mediation of other’s intentions relative to females [62]. Lai and colleagues [63] also demonstrated that the cerebellum has been linked to sex-related conditions and traits including autism spectrum traits. Taken together, our findings suggest that increased fALFF and ReHO in the IFG and cerebellum may be a link to a male-related pattern of DMN. They also appear to indicate that, females and males differ with regard to local connectivity and synchronicity of spontaneous brain activity in the DMN in relation to their autism spectrum traits.

Previous task-related fMRI studies have reported that brain regions including the ANG [55], MTG [64], TempP [65], SFG [66], MOG [67], MPFC [25,55–57], and OFC [68] are associated with social cognition processing. Our results showed significant differences in functional connectivity and regional measurements within several of these brain regions in male subjects. This suggests that sex differences in the DMN may be strongly associated with social cognition processing. More specifically, we speculate that that sex-related differences in the regional activation and connectivity of the DMN might be linked to social cognition processing. Interestingly, previous study also show a significant group difference in imagination scores on the AQ, which have been shown to interact the broad autism phenotype (BAP) in previous [69], warranting further investigation.

### Sex differences in the DMN associated with autism spectrum traits

We found a strong negative correlation between AQ social interaction scores and the strength of the rs-FC in the aMPFC and TempP in males but not in females. Our findings suggest that the strength of the rs-FC of the DMN may account for the level of autism spectrum traits in males, thereby contributing to the characteristic differences from females [70]. Olson et al. [65] demonstrated that the TempP plays a pivotal role in both social and emotional processes including face recognition and theory of mind, which are important factors determining the extent of autism spectrum traits. These findings, together with our current results, provide support for the extreme male brain theory by showing how autism spectrum traits differentially affect the male brain [13,14,63]. Collectively, the existing evidence indicates that the male brain exhibits an imbalance in the DMN that is associated with autism spectrum traits. We hypothesize that this skewed balance may be a potential biomarker to objectively measure the level of extreme male brain characteristics.

In the current study, our findings may be preliminary neurobiological evidence of a relationship between the DMN and autism spectrum traits in typical males, based on extreme male brain theory. However, most studies of ASD do not include enough females to conduct gender comparison, or recruit only males; this opens up the possibility that our knowledge of extreme male brain theory is biased by a limited knowledge of the female profile. Additional research targeting females with and without SD would help expand our understanding of autism spectrum traits and the need for clinical support regardless of gender.

### Future directions

Although our results may help clarify the relationship between sex differences in the DMN and autism spectrum traits, whether our results can be generalized to different age ranges is still unclear. Future studies will need to replicate these findings in children for a more comprehensive understanding of sex differences, ASD and normal development. A large-scale study of human sex differences in autism spectrum traits points to more-systemizing-less-empathizing features in males based on results of the Empathy Quotient, Systemizing Quotient-Revised (SQ-R), and AQ [14]. Additional studies that employ these three tests are needed to provide more support for an extreme male brain theory.

A potential limitation of this study is that there was no formal assessment of intelligence of the participants. As such, we cannot be sure whether IQ may have played a role in our results, mediating functional connectivity between regions. Therefore, more work is needed to ascertain if these findings can be reproduced in males and females matched on intelligence. Additional studies are also needed in order to confirm the relationship between the DMN and autism spectrum traits with the BAP.

Finally, rs-fMRI provides information about functional connectivity in the human brain at rest, free from task effects. One study indicated that different levels of anxiety and arousal might affect resting functional connectivity at rest [71], necessitating an examination of these effects in males and females.

### Conclusions

Our results show that young males have different fALFF, ReHo, and connectivity characteristics in the DMN compared with age-matched females. In addition, we showed that the strength of the connections in default-mode resting state network was associated with autism spectrum traits in male subjects only. Our findings provide insight into the skewed balance of functional connectivity in the DMN and suggest that this parameter may be a potential biomarker to objectively identify autism spectrum traits within the context of the extreme male brain theory.

## Supporting Information

S1 Dataset The demographic, AQ scores. (PDF)
