# Supplementary material for: Sex Differences in the Default Mode Network with Regard to Autism Spectrum Traits: A Resting State fMRI Study
Source: PLoS One. 2015 Nov 24;10(11):e0143126. doi: 10.1371/journal.pone.0143126 (PMC4658035; doi:10.1371/journal.pone.0143126)
Supplement: S1 Dataset — (PDF) [file pone.0143126.s001.pdf]

Male	AGE	AQ-S	AQ-AS	AQ-AD	AQ-C	AQ-I	AQTotal
M01	19	5	3	5	2	3	18
M02	22	2	7	5	4	5	23
M03	22	3	5	6	4	3	21
M04	19	8	6	7	7	3	31
M05	22	0	3	2	0	4	9
M06	23	2	6	2	3	5	18
M07	32	2	5	3	1	2	13
M08	21	3	6	4	1	2	16
M09	30	6	6	4	3	6	25
M10	22	0	5	4	0	3	12
M11	31	2	4	7	1	7	21
M12	25	2	2	8	0	3	15
M13	21	1	4	0	1	1	7
M14	20	5	6	3	3	4	21
M15	22	3	5	1	2	6	17
M16	22	3	6	2	2	6	19
M17	24	6	7	5	6	4	28
M18	23	3	2	4	2	4	15
M19	21	5	4	5	3	2	19
M20	27	7	4	4	4	4	23
M21	27	3	6	3	7	3	22
M22	26	3	4	4	1	3	15
M23	35	5	6	2	6	4	23
M24	20	1	4	2	2	1	10
M25	26	5	4	2	2	4	17
M26	27	5	1	3	3	3	17
M27	22	4	4	0	2	3	13
M28	22	2	4	6	3	1	16
M29	21	1	6	3	3	3	16
M30	27	3	7	2	2	4	18
M31	22	4	6	4	2	2	18
M32	19	1	3	3	0	3	10
M33	25	2	0	4	0	3	9
M34	23	6	4	4	4	0	18
M35	31	4	5	0	4	4	17
M36	25	2	2	2	0	2	8
M37	29	4	5	6	4	4	23
M38	25	4	3	4	0	6	17
M39	21	3	3	5	6	3	20
M40	20	2	3	5	2	4	16
M41	20	7	7	6	4	6	30
M42	21	1	0	4	0	1	6
M43	20	2	5	7	2	4	20
Mean	**23.8**	**3.3**	**4.4**	**3.8**	**2.5**	**3.5**	**17.5**

Female	AGE	AQ-S	AQ-AS	AQ-AD	AQ-C	AQ-I	AQTotal
F01	21	0	1	2	1	1	5
F02	22	2	3	4	3	4	16
F03	22	1	4	2	3	1	11
F04	28	0	3	3	0	2	8
F05	25	3	0	0	2	5	10
F06	24	4	3	4	2	3	16
F07	22	6	7	5	6	3	27
F08	39	2	4	1	2	4	13
F09	20	5	2	1	6	4	18
F10	24	5	5	4	3	2	19
F11	27	2	2	1	0	0	5
F12	24	8	7	4	9	4	32
F13	22	0	3	3	3	1	10
F14	20	0	4	10	2	2	18
F15	30	7	9	7	7	2	32
F16	20	1	2	8	1	2	14
F17	22	0	2	6	2	1	11
F18	28	5	2	1	6	1	15
F19	21	4	3	5	2	1	15
F20	20	4	5	5	3	2	19
F21	22	4	5	3	2	2	16
F22	22	3	7	3	1	2	16
F23	22	2	6	2	2	2	14
F24	20	3	5	2	4	2	16
F25	21	6	6	8	6	1	27
F26	19	7	8	3	5	2	25
F27	21	3	4	4	1	1	13
F28	20	0	5	2	0	3	10
F29	20	4	7	1	3	2	17
F30	18	2	6	6	2	2	18
F31	20	0	3	3	1	1	8
F32	18	1	4	6	0	2	13
F33	18	0	1	8	0	2	11
F34	18	0	6	2	5	2	15
F35	22	1	6	4	3	1	15
F36	22	3	6	2	4	2	17
F37	25	1	2	3	2	2	10
F38	21	1	3	3	2	3	12
F39	18	0	3	2	0	0	5
F40	18	0	3	7	0	1	11
F41	22	6	3	2	4	1	16
F42	32	1	4	1	2	0	8
Mean	**22.4**	**2.5**	**4.1**	**3.6**	**2.7**	**1.9**	**15.0**
